# A role for human leucocyte antigens in the susceptibility to SARS‐Cov‐2 infection observed in transplant patients

**DOI:** 10.1111/iji.12505

**Published:** 2020-07-05

**Authors:** Kay Poulton, Paul Wright, Pamela Hughes, Sinisa Savic, Matthew Welberry Smith, Malcolm Guiver, Muir Morton, David van Dellen, Eleni Tholouli, Robert Wynn, Brendan Clark

**Affiliations:** ^1^ Transplantation Laboratory Manchester Royal Infirmary Manchester UK; ^2^ Manchester Academic Health Science Centre University of Manchester Manchester UK; ^3^ Transplant Immunology St James’s University Hospital Leeds UK; ^4^ Department Immunology St James’s University Hospital Leeds UK; ^5^ Department Renal Medicine St James’s University Hospital Leeds UK; ^6^ Department Virology Manchester Royal Infirmary Manchester UK; ^7^ Department Renal Medicine Manchester Royal Infirmary Manchester UK; ^8^ Department Renal Transplantation Manchester Royal Infirmary Manchester UK; ^9^ Department Haematology Manchester Royal Infirmary Manchester UK; ^10^ Paediatric BMT Unit Royal Manchester Children’s Hospital Manchester UK

**Keywords:** genetics, genetics, case/control studies, histocompatibility, immune response, human leucocyte antigens, immunology, disease association studies, molecular, population, severe acute respiratory syndrome, virology, disease association, virus

## Abstract

We analysed data from 80 patients who tested positive for SARS‐CoV‐2 RNA who had previously been HLA typed to support transplantation. Data were combined from two adjacent centres in Manchester and Leeds to achieve a sufficient number for early analysis. HLA frequencies observed were compared against two control populations: first, against published frequencies in a UK deceased donor population (*n* = 10,000) representing the target population of the virus, and second, using a cohort of individuals from the combined transplant waiting lists of both centres (*n* = 308), representing a comparator group of unaffected individuals of the same demographic. We report a significant HLA association with HLA‐ DQB1*06 (53% vs. 36%; *p* < .012; OR 1.96; 95% CI 1.94–3.22) and infection. A bias towards an increased representation of HLA‐A*26, HLA‐DRB1*15, HLA‐DRB1*10 and DRB1*11 was also noted but these were either only significant using the UK donor controls, or did not remain significant after correction for multiple tests. Likewise, HLA‐A*02, HLA‐B*44 and HLA‐C*05 may exert a protective effect, but these associations did not remain significant after correction for multiple tests. This is relevant information for the clinical management of patients in the setting of the current SARS‐CoV‐2 pandemic and potentially in risk‐assessing staff interactions with infected patients.

## 1 BACKGROUND

The capacity of an individual to mount an effective immune response to infection is entirely dependent upon their inherited complement of human leucocyte antigens (HLA) which present pathogenic peptides to T and B lymphocytes. The repertoire of the viral peptides bound by each individual is dependent upon sequence variation within the antigen recognition site of their HLA molecules. This has recently been underlined in the twin study of Williams et al. (2020). The HLA type of the patient is likely to be a significant influencing factor in an infected individual's ability to produce an effective immune response (Ishibashi et al., 2009). A number of in silico studies have defined the potential for immune responsiveness based on differential viral peptide binding to HLA alleles (Fast, Altman, & Chen, 2020; Nguyen et al., 2020). These are useful in vaccine design but do not represent the situation in natural infection where the full HLA type of the individual, comprising up to twelve alleles across six loci, informs the capacity for immune response. This study investigated HLA profiles of patients admitted with PCR‐confirmed SARS‐CoV‐2 infection to identify any potential HLA bias which might indicate an impaired capacity to mount an effective immune response to the infection. The information is of value towards risk stratification and clinical management of patients and potentially in risk‐assessing staff interactions with infected patients.

## 2 METHODOLOGY

The aim of this study was to analyse the HLA types of individuals receiving hospital care for COVID‐19 symptoms, where the HLA type was already known. The inclusion criteria for this study were (a) that the patient must have been previously HLA typed at least at HLA‐A, B, C, DRB1 and DQB1 and (b) must have a positive test for the presence of SARS‐CoV‐2 RNA. A total of 80 patients were included in the study, 41 from Manchester and 39 from the Leeds transplant centre. Data were combined from both units to achieve a sufficient number for early analysis. All patients included in the study had previously been HLA typed to support transplantation and required hospital treatment for COVID‐19 disease, indicating that their symptoms were severe, requiring clinical support or intervention. Patients included in the study were either kidney transplant patients (*n* = 33), individuals on the solid organ transplant waiting lists (*n* = 40), or patients who had received haematopoietic stem cell transplantation (*n* = 7). For the Leeds patients, the male:female ratio was 32:8, and the median age was 60.6 years (range 27–87 years). For the Manchester patients, the male:female ratio was 20:20, and the median age was 58.9 years (range 3–80 years).

HLA typing had been performed using either of two methods: (a) LABType^®^SSO HLA Class I (HLA‐A, B and C) and HLA Class II (DRB1/3/4/5 and HLA‐DQ) loci (OneLambda Inc), or (b) next‐generation sequencing (HOLOTYPE HLA™; Omixon or TruSight HLA, Illumina). All tests were performed according to manufacturers’ instructions. HLA frequencies were analysed at first field resolution, and where second field typing data were available, types were converted into a serological equivalent specificity for analysis.

## 3 CONTROL POPULATIONS

We have performed analyses against two control populations Group A: a UK (*n* = 10,000) deceased donor pool13 (https://nhsbtdbe.blob.core.windows.net/umbrarco‐assets‐corp/2925/antigen.pdf)and Group B: a local (*n* = 308) wait‐listed renal patient cohort comprising the active wait list from Leeds (*n* = 150) and a randomly selected cohort (*n* = 158) from the Manchester centre. The rationale behind the use of two separate control groups was an acknowledgement that problems exist in respect of both. Control Group A is representative of the UK population and the infection target of the virus, but it lacks proportionate representation of Black, Asian and Minority Ethnic (BAME) groups. Comparisons made with transplant patient groups may be confounded by this variance. The advantage of using the control Group A in the context of this study is, however, that it represents a population assembled prior to the SARS‐CoV‐2 pandemic. Conversely, the *n* = 308 cohort (Group B) is derived by combining unselected individuals on the renal transplant waiting list from each centre, but the SARS‐CoV‐2 infection/exposure history of the group is uncertain and incorrect inferences may then be drawn from its use. Alternative control groups were considered but precluded on the basis of the problems in their confident construction at this time.

### 3.1 Statistical analysis

Statistical analysis was performed using MedCalc v19.3 (MedCalc Software) to compare frequencies of allele group carriage between the patient and control populations using Fisher's exact test. Odds ratios were calculated at 95% confidence intervals (CI) using MedCalc software. Each allele group was compared independently, and *p* ≤ .05 was considered to be significant. Bonferroni correction was applied to accommodate multiple testing within each locus tested (Gaetano, 2018). The allelic frequencies were assessed in all populations.

Allele group carriage is defined as the number of individuals carrying the respective allele group. Allelic frequencies are defined as the number of occurrences of the respective allele group divided by the total number of allele groups in the cohort. An allele group was considered to raise the risk of viral susceptibility when the OR was above 1 and considered protective against viral susceptibility when OR was below 1.

## 4 RESULTS

Allele group carriage frequencies for all specificities where associations which were significant, or approaching significance are included in Table 1. Seventeen of the top twenty most frequent HLA haplotypes in the UK population (allelefrequencies.net) were represented in this cohort revealing a broad susceptibility to infection. When comparing our patients with control Group A, there were more significant associations observed than when compared against control Group B. The strongest associations observed in risk of viral susceptibility observed were with HLA Class II specificities. After Bonferroni correction, HLA‐DQB1*06 remains a significantly increased risk when compared with Group B controls, but not Group A controls (Group A: 52.5% vs. 41.0%; *p* = .0402, *p*_c_ = not significant; OR = 1.59 CI 1.02–2.47; *p* = .0390, *p*_c_ = not significant; Group B: 52.5% vs. 36.0%; *p* = .0100, *p*_c_ = .0523; OR = 1.96 CI 1.19– 3.22; *p* = .0078; *p*_c_ = .0468.) Additionally, there is a significant bias in this group of patients which did not retain significance after the application of Bonferroni correction with higher‐than‐expected frequencies of HLA‐DRB1*15 (Group A: 40.0% vs. 28.0%, *p* = .0238; OR = 1.71 CI 1.09–2.68, *p* = .0187; Group B: 40.0% vs. 25.3%, *p* = .0121; OR = 1.97 (1.17–3.29), *p* = .0102) and DRB1*11 (Group A: 22.5% vs. 13.0%, *p* = .0185; OR = 1.94 CI 1.15–3.29, *p* = .0137; Group B: 22.5% vs. 18.8%, *p* = not significant) in our patient group. The antigens comprising the HLA‐DQ1 group (HLA‐DQB1*05 and DQB1*06), which are in tight linkage disequilibrium with HLA‐DRB1*15 and HLA‐DRB1*10, were collectively of raised risk (Group A: 72.5% vs. 58.0%; *p* = .0087, *p*_c_ = .0523; OR = 1.91 CI 1.17–3.12; Group B: 72.5% vs. 60.1%; *p* = .0513, *p*_c_ = not significant; OR = 1.96 CI 1.19–3.22, *p* = .0421, *p*_c_ = not significant). When the broad specificities were considered independently, HLA‐DQB1*05 was not significant in its correlation with viral infection, suggesting that the primary association lies with DQB1*06, rather than DQ1. With such a small cohort, it is not possible to ascertain whether these observations are due to linkage disequilibrium between HLA‐DR and DQ, or whether this is an independent observation. For HLA Class I, HLA‐A*26 was also significantly increased in the patients compared with Group A controls (11.3% vs. 4.0%; *p* = .0049, *p*_c_ = .0198; OR = 3.04 CI 1.50–6.13, 0.0019, *p*_c_ = .0076). These data need to be interpreted cautiously as the associations reaching significance against Group A controls are based on comparison with a UK population and HLA‐DRB1*10 and HLA‐A*26 are known to be represented at higher frequency in the BAME populations. Neither of these associations remained significant when compared with the local population of Group B controls.

**Table 1:** HLA frequencies in 80 patients with COVID‐19 previously HLA typed to support transplantation

HLA Allele Group	COVID‐19 population Allele group carriage frequency (%; *n* = 80)	Control Group A Allele group carriage frequency (%; *n* = 10,000)	Control Group A *p*	Control Group A *p*_c_	Control Group B Allele group carriage frequency (%) (*n* = 308)	Control Group B *p*	Control Group B *p*_c_
HLA‐A*02	36.3	50.0	.0179	.0536	46.4	—	—
HLA‐A*26	11.3	4.0	.0049	.0198	7.5	—	—
HLA‐B*44	17.5	32.0	.0052	.0105	19.8	—	—
HLA‐C*05	8.8	21.0	.0054	.0215	15.9	—	—
HLA‐C*12	12.5	6.0	.0286	—	11.7	—	—
DRB1*15	40.0	28.0	.0238	—	25.3	.0121	—
DRB1*04	22.5	35.0	.0185	—	33.4	—	—
DRB1*11	22.5	13.0	.0185	—	18.8	—	—
DRB1*07	16.3	26.0	.0539	—	20.8	—	—
DRB1*10	6.3	1.0	.0014	.0144	4.5	—	—
DQB1*05	35.0	27.0	—	—	30.5	—	—
DQB1*06	52.5	41.0	.0402	—	36.0	.0010	.0523

Note

The control frequencies are derived from Group A: (*n* = 10,000) deceased solid organ donors between November 1994 and December 2009 (https://nhsbtdbe.blob.core.windows.net/umbraco‐assets‐corp/2925/antigen.pdf) and Group B: a local (*n* = 308) wait‐listed renal patient cohort comprising the active wait list from Leeds (*n* = 150) and a randomly selected cohort (*n* = 158) from the Manchester centre. Only associations which demonstrated significance, or approached significance when compared with either control population are listed. Significance (Fisher's exact) is shown before (*p*) and after (*p*_c_) Bonferroni correction. Results which are not significant are represented by (−) in the table.

A number of HLA alleles appeared to exert a protective effect when compared with the Group A controls and were identified at lower‐than‐expected frequencies in our patient population. For example, HLA‐A*02 (36.3% vs. 50.0%, *p* = .0179, *p*_c_ = .0536; OR = 0.57 CI 0.36–0.90, *p* = .0156, *p*_c_ = .0468), B*44 (18% vs. 32%, *p* = .0052, *p*_c_ = .0105; OR = 0.45 CI 0.25–0.80, *p* = .0069, *p*_c_ = .0138)C*05 (9% vs. 21%, *p* = .0054, *p*_c_ = .0215; OR = 0.36 CI 0.17–0.78, *p* = .0101, *p*_c_ = .0404), DRB1*04 (22.5% vs. 35.0%, *p* = .0185, *p*_c_ = not significant; OR = 0.54, CI 0.32–0.91, *p* = .0214, *p*_c_ = not significant) and DRB1*07 (16.3% vs. 26.0%; *p* = .0539, *p*_c_ = not significant; OR = 0.55, CI 0.30–1.00; *p* = .0507, *p*_c_ = not significant). It is of interest that HLA‐A*02, HLA‐B*44 and HLA‐C*05 are frequently inherited together with either DRB1*04 or HLA‐DRB1*07 in English population (Allele Frequency Net Database allelefrequencies.net) which may reinforce the possibility that a protective effect, or a superior immune response to SARS‐CoV‐2 infection is exerted by these ancestral haplotypes.

## 5 DISCUSSION

Effective immune clearance of any virus can only be achieved when an individual's HLA antigens collectively succeed in presenting pathogen‐derived peptide efficiently, enabling the generation of sufficient antibody to eliminate it. Without this, a situation arises in which the reproduction of virus within the host results in ongoing invasion, overwhelming the capacity of the affected individual to effectively clear the infection. In SARS‐CoV‐2 infection, a high‐affinity neutralizing antibody response would act in synergy with the production within the lung of antiviral cytokines and promotion of the CD8 T‐cell response (DiPiazza, Richards, Knowlden, Nayak, & Sant, 2016).

Individuals infected with SARS‐CoV‐19 have a variety of clinical manifestations of the disease described as COVID‐19 (Huang et al., 2020). While almost all patients develop COVID pneumonia, approximately 29% cases progress to acute respiratory distress syndrome which occurs 10–12 days after the onset of symptoms (Huang et al., 2020). Furthermore, individuals who develop respiratory distress have often been responding well during the first phase of the disease, but their symptoms return aggressively, requiring clinical support within a hospital environment. This phenomenon is suggestive of an immunological hiatus, in which these individuals have been unable to effectively mature their immune response to generate the production of potent neutralizing antibody.

In this series of individuals requiring admission following infection with SARS‐CoV‐2, the possibility presents that possession of the HLA Class I type HLA‐A*26 or the HLA Class II types HLA‐DQB1*06, HLA‐DRB1*15 or HLA‐DRB1*10 results in an impaired ability to present the viral peptides necessary to form a protective T‐cell repertoire. An alternative explanation may be that the HLA molecule itself is implicated as an attachment factor for the spike protein as documented by Chan et al. (2009) in coronavirus HKU1 infection, where evidence suggests that HLA‐C molecules actually facilitate the entry of the virus into the cell.

In this regard, HLA‐A*26 has previously been identified as a predisposing type to infection with visceral leishmaniasis (kala‐azar; Singh, Agrawal, & Rastogi, 1997) and has been associated with the development of Epstein–Barr virus‐driven post‐transplant lymphoproliferative disorder in solid organ transplantation (Reshef et al., 2011). The occurrence of other specificities potentially within the HLA‐A*26 superset is also noteworthy and is the subject of further work in our laboratories. We also note the prediction, based on an in silico analysis, for poor ability of the closely related antigen HLA‐A*25 to present a repertoire of SARS‐CoV‐2 epitopes in the current paper from Nguyen et al. (2020). This paucity would be expected to impact on the quality of the linked immune response to the virus. Utilizing the Immune Epitope Database Analysis resource, TepiTool (TepiTool IEDB Analysis Resource), we therefore examined the top potential epitopes (Fast et al., 2020) for key SARS‐Cov‐2 proteins in regard to their binding affinity to HLA‐A*26:01. Based on IC50 < 500 nm, only one epitope (FTISVTTE) was identified that would be predicted to support a T‐cell‐based immune response. This link between a real‐world observation and an in silico predication provides validation of both findings. In this study, the individuals who were positive for HLA‐A*26 were not also positive for HLA‐DRB1*10, although these have been documented to be inherited on the same haplotype (allelefrequencies.net).

A study of SARS‐CoV‐2‐positive individuals in a UK Biobank population has also identified DRB1*15:01 (OR = 1.33, CI 1.01–1.74) and DQB1*06:02 (OR = 1.32, CI 1.01–1.74) to be associated with an increased likelihood of testing positive for the virus (Kachuri et al., 2020). This is an in silico study in which HLA types have been imputed, but these observations add support to our own observation in regard to HLA‐DR15 and DQ6 in this early study of individuals with known HLA types. The upregulation of HLA‐DR expression in lung tissue of patients affected with COVID pneumonia (Wang et al., 2020) suggests that this process is instrumental in the immunological control of the disease process and therefore must be considered to be biologically relevant. HLA‐DR10 and DR11(5) have previously been implicated in patients with impaired response to cytomegalovirus infection (Ishibashi et al., 2009), while HLA‐DR11(5) has also been documented to be associated with severe response to infection with MERS‐Cov (Hajeer, Balkhy, Johani, Yousef, & Arabi, 2016). HLA‐DR10 has also been proposed to be the vector mechanism for viral entry of Epstein–Barr virus into B cells, reinforcing the concept that the molecule itself may be a mechanism for driving viral infection Li and Cohen (2019). A primary inefficiency of the alleles HLA‐DQB1*05 and *06 cannot be discounted in this very early UK study, due to linkage disequilibrium.

This study has been deliberately independent of ethnicity as the UK population in this region of England is genetically heterogeneous with multiple populations of different heritages co‐existing. It could be argued that the use of the UK deceased donors as a control population in this study has simply highlighted more frequent occurrence of antigens common to BAME communities in our COVID‐19 population requiring hospital support for their disease. Conversely, it may be legitimately argued that the BAME link with susceptibility is explained through possession of these antigens. While either point remains valid, other specificities common to BAME groups such as HLA‐A*34 and DRB1*16 were not implicated as having heightened susceptibility to severe infection in this study. HLA‐DRB1*10 and A*26 are common within all European subregions (Sanchez‐Mazas et al., 2017) and offer an independent mechanistic explanation for severity of clinical disease in COVID‐19 patients which is independent of skin colour or other immunogenetic variation due to heritage. If a wider study were to be conducted in which all individuals positive for SARS‐CoV‐2 RNA could be included, encompassing all ranges of clinical severity from mild symptoms to severe infection, it could be predicted that the increased frequency of HLA‐DRB1*10 and A*26 in individuals with the most clinically detrimental symptoms could take on greater significance. The identification of these alleles as factors informing disease susceptibility underlines the importance of further consideration of HLA and other immunogenetic influences in ongoing efforts to address the SARS‐Cov‐2 pandemic.

These are obviously tentative findings based on a limited number of cases in two centres. An unbiased surveillance of the transplant population to include those with mild symptoms or no symptoms would help to clarify this observation. We are now seeking to accrue additional data in substantiation of the association and to extend the scope of our study to in order to establish its basis.
